# Small nucleolar RNA host gene 1 promotes development and progression of colorectal cancer through negative regulation of miR‐137

**DOI:** 10.1002/mc.23101

**Published:** 2019-08-30

**Authors:** Yang Fu, Yuhan Yin, Sanfei Peng, Ge Yang, Yang Yu, Changqing Guo, Yanru Qin, Xiefu Zhang, Wen Xu, Yiyu Qin

**Affiliations:** ^1^ Department of Gastroenterology The First Affiliated Hospital of Zhengzhou University Zhengzhou Henan China; ^2^ Department of Ophthalmology The First Affiliated Hospital of Zhengzhou University Zhengzhou Henan China; ^3^ Department of Oncology The First Affiliated Hospital of Zhengzhou University Zhengzhou Henan China; ^4^ State Key Laboratory of Bioreactor Engineering & Shanghai Key Laboratory of New Drug Design, School of Pharmacy East China University of Science and Technology, Shanghai Shanghai China; ^5^ Research Centre of Biomedical Technology Jiangsu Vocational College of Medicine Yancheng Jiangsu China

**Keywords:** colorectal cancer, miR‐137, rapamycin‐insensitive companion of mTOR, small nucleolar RNA host gene 1

## Abstract

Small nucleolar RNA host gene 1 (SNHG1) is critical in the progression of cancers. However, the mechanism by which SNHG1 regulates the progression of colorectal cancer (CRC) remains unclear. Expressions of SNHG1 and miR‐137 in CRC tissues and cell lines were evaluated by quantitative real‐time polymerase chain reaction. A luciferase reporter gene assay was conducted to investigate miR‐137 target. Additionally, RNA pull‐down assay was performed to explore the physical association between miR‐137, SNHG1, and RNA induced silencing complex (RISC). Cell cycling and invasion were examined by flow cytometry (FCM) and transwell assays. The in vivo carcinogenic activity of SNHG1 was examined using murine xenograft models. Expression of RICTOR, serine/threonine kinase 1 (AKT), serum and glucocorticoid‐inducible kinase 1 (SGK1), p70S6K1, and LC3II/LC3I ratio was examined by Western blot analysis. SNHG1 upregulation was observed in CRC tissues and cell lines, which was associated with the lymph node metastasis, advanced TNM stage and poorer prognosis. SNHG1 increased RICTOR level in CRC via sponging miR‐137. In addition, SNHG1 silencing inhibited CRC cell proliferation and migration in vitro and in vivo. SNHG1 regulated RICTOR expression by sponging miR‐137 and promoted tumorgenesis in CRC.

**Abbreviations**
3′‐UTR: 3′‐untranslated region
AKT: serine/threonine kinase 1
CRC: colorectal cancer
DMEM: Dulbecco's modified Eagle's medium
FCM: flow cytometry
IHC: immunohistochemistry
MUT: mutant
NC: negative control
ncRNAs: noncoding RNAs; HMGA2
p70S6K1: ribosomal protein S6 kinase, 70 kDa
PBST: phosphate‐buffered saline with Tween‐20
qRT‐PCR: quantitative real‐time polymerase chain reaction
RICTOR: rapamycin‐insensitive companion of mTOR
RISC: RNA induced silencing complex
SGK1: serum and glucocorticoid‐inducible kinase 1
SNHG1: small nucleolar RNA host gene 1
WT: wild‐type

## 1 INTRODUCTION

Colorectal cancer (CRC) is the third most common malignant tumor in humans, and is also the most common malignant gastrointestinal tumor.^1^ Although considerable efforts have been made to clarify the etiology and pathogenesis of CRC, many questions remain unanswered.^2^, ^3^ It is important to elucidate the molecular mechanisms of CRC tumorigenesis and progression to facilitate the development of effective treatments.

The Human Genome Project has demonstrated that less than 2% of the human genome consists of protein‐coding genes, while greater than 90% produces noncoding RNAs (ncRNAs).^4^ The ncRNA family can be divided into many categories—according to size, structure, and function—including microRNAs (miRNAs) and long noncoding RNAs (lncRNAs). Many studies have focused on ncRNA‐mediated protein‐coding gene regulation, overall indicating that ncRNAs form an interaction network which fine‐tunes regulation of gene expression. More recently, the competing endogenous RNA (CeRNA) hypothesis was proposed. This suggests that a large number of ncRNA types may interact with and sequester miRNAs, thereby derepressing the function of alternate and unsequestered transcripts of these miRNAs, providing a novel mechanism contributing to posttranscriptional regulation of target genes.^5^, ^6^ Moreover, aberrant expression of ncRNAs has been documented in several human tumors, suggesting that ncRNAs may significantly contribute to their pathogenesis.^7^, ^8^ For example, lncRNA‐HULC overexpression may suppress miR‐186 expression, resulting in increased expression of its target protein, HMGA2, which serves as an oncogene in hepatocellular carcinoma.^9^ Such mRNA/miR‐lncRNA coregulatory pairs (eg, TCF7L2/miR‐217‐CRNDE and TUSC7/miR‐211‐CDK6) have also been demonstrated in CRC.^10^, ^11^

SNHG1, located on chromosome 11q12.3, is 1134 base pairs (bps) long and its intronic sequence encodes eight small nucleolar (sno) RNAs: SNORD22 and SNORD25‐31.^12^ Previous studies suggested that abnormal upregulation of SNHG1 was observed in lung cancer, liver cancer, and neuroblastoma, which was negatively correlated with prognosis.^13^, ^14^, ^15^ It has been documented that SNHG1 promoted the growth of primary esophageal cancer, hepatocellular carcinoma, and non‐small–cell lung cancer via sponging several tumor‐suppressive miRNAs including miR‐338, miR‐195, and miR‐101‐3p.^12^, ^16^, ^17^ Based on the above results, SNHG1 possesses the characteristics of an oncogene in many tumor types; however, the role of SNHG1 in CRC remains unclear.

## 2 MATERIALS AND METHODS

### 2.1 Clinical samples

Written informed consent was obtained from all participants (CRC patients). This study protocol complied with the Declaration of Helsinki and was approved by the Human Ethics Committee of the First Affiliated Hospital of Zhengzhou University. A total of 80 patients with CRC who underwent radical resection (2011/4‐2013/5) were enrolled from the General Surgery Department of the First Affiliated Hospital of Zhengzhou University (Zhengzhou, China). Tumor and adjacent normal tissues were collected, and each sample was immediately snap‐frozen in liquid nitrogen and then stored at −80℃ before RNA extraction for quantitative real‐time polymerase chain reaction (qRT‐PCR) analysis. All study participants had received no additional preoperative therapy, and underwent TNM staging using criteria adopted from the American Joint Committee on Cancer/Union for International Cancer Control Rectal Cancer TNM Staging System (7th Edition, 2010).^18^ Detailed clinical (including follow‐up) data were collected for each patient.

### 2.2 Cell culture

Human colorectal carcinoma cell lines LoVo, HT‐29, T84, and HCT116, and a human embryonic kidney cell line, HEK293T, were purchased from the Cell Bank of the Chinese Academy of Sciences (Shanghai, China), and an immortal normal colorectal epithelial cell line, HCoEpic, was purchased from Sciencell Research Laboratories (SRL; Carlsbad, CA). All cell lines were cultured in complete Dulbecco's modified Eagle's medium (DMEM) (Gibco BRL, Grand Island, New York) supplemented with 10% fetal bovine serum (Gibco BRL) and 1% penicillin/streptomycin (Invitrogen, Carlsbad, CA), at 37°C in a humidified incubator (5% CO_2_ atmosphere). All cell lines were cultured for at least 6 months.

### 2.3 Quantitative real‐time polymerase chain reaction

The expression levels of SNHG1, miR‐211, miR‐137 and other potential target miRNAs of SNHG1 such as miR‐101‐3p, miR‐4735‐3p were examined by qRT‐PCR. Total RNA was extracted from cultured cells using TRIzol reagent (Invitrogen) according to the manufacturer's instructions. Isolated RNA was reverse‐transcribed to produce complementary DNA (cDNA) utilizing the PrimerScript one‐step RT‐PCR kit (Takara, Dalian, People's Republic of China). The SYBR Premix Dimmer Eraser on the StepOnePlus Real‐Time PCR System (Takara) was used for qRT‐PCR analysis. PCR proceeded as follows: 95°C for 300 seconds; 35 cycles at 95°C for 10 seconds, 60°C for 30 seconds, and 75°C for 15 seconds. Expression of all genes was normalized to glyceraldehyde 3‐phosphate dehydrogenase (GAPDH) using $2^{-\Delta \Delta C_{t}}$ methods.^19^ Each qRT‐PCR reaction was conducted in triplicate. Primer sequences are provided in the Supporting Information Materials.

### 2.4 Quantitation of mature miRNA levels using qRT‐PCR

The Starbase V2.0 software (Sun Yat‐sen University, Guangzhou, China) was used to predict the target miRNAs of SNHG1, and the URL was listed as follows: MEP_L_bib20. Primers were designed according to miRNA mature sequences. Total cell RNA was extracted using TRIzol reagent, and then reverse‐transcribed into cDNA using the Prime Script miRNA cDNA Synthesis Kit (Takara). Poly A was added to the 3′ end of miRNA and used for reverse transcription by the primer sequence of the oligonucleotide (dt). PCR proceeded as follows: 95°C for 30 seconds, followed by 35 cycles of 95°C for 10 seconds, 60°C for 30 seconds, and 72°C for 15 seconds. Relative transcript levels were quantified using the $2^{-\Delta \Delta C_{t}}$ method. An internal reference (snRNA U6) was included. Each qRT‐PCR reaction was conducted in triplicate.

### 2.5 Cell transfection

Hsa‐miR‐137 mimic/negative control mimic and Hsa‐miR‐137 inhibitor/negative control were purchased from Shanghai Genechem Co, Ltd (Shanghai, China). Two small interfering RNAs (siRNAs) targeting SNHG1 (si‐SNHG1‐1 and ‐2) were purchased from the Shanghai Sangon Company (Shanghai, China) and two siRNAs targeting RICTOR (si‐RICTOR1 and ‐2) were purchased from Santa Cruz Biotechnology (Santa Cruz, CA). Transfection was carried out using a Lipofectamine 2000 Kit (Invitrogen). After PCR‐based amplification of SNHG1, this gene was cloned into the *Hind*III and *EcoR*I sites of a pcDNA3.1 vector, then renamed pcDNA3.1‐SNHG1. Additionally, miR‐137 response elements (MREs) were introduced using the Quik Change Site‐Directed Mutagenesis Kit (Stratagene, La Jolla, CA). After the introduction of MRE point mutations, the vector was renamed pcDNA3.1‐ SNHG1 mutant.

The potential miRNA binding sites of SNHG1 or SNHG1 mutants were amplified and subcloned into a pmirGLO vector to facilitate luciferase reporter gene analysis. The RICTOR mRNA3′‐untranslated region (UTR)‐containing an intact miR‐137‐family recognition sequence was also amplified and subcloned into the *Sac*I and *Sa*lI sites of a pmirGLO vector.

Cells were grown in six‐well plates to 60% confluence, transfected using Lipofectamine 2000 according to the manufacturer's instructions), and harvested after 48 hours for Western blot analysis or qRT‐PCR. Final concentrations of miRNAs/plasmids during the assays were as follows: RICTOR siRNA/negative control siRNA 20 nM/mL, SNHG1‐wild‐type (WT)/SNHG1‐mutant (MUT) 50 nM/mL, miR‐137 mimic/negative control (NC) 100 nM/mL, and miR‐137 inhibitor 200 nM/mL.

### 2.6 Luciferase reporter gene assay

Using pmirGLO, pmirGLO‐SNHG1 wt, and pmirGLO‐SNHG1 mut, along with miR‐137 mimics, LoVo and HT‐29 cells were cotransfected (method as described above). After 48 hours of transfection, luciferase activity assays were performed using a Secrete‐Pair Dual‐Luminescence Assay Kit (GeneCopoeia, Rockville, MD). Results were obtained from three independent experiments, each performed in duplicate.

### 2.7 RNA pull‐down assay

To examine whether SNHG1 was bound to the RISC, an RNA pull‐down assay was carried out as previously described.^11^ Briefly, SNHG1‐wt fragments were synthesized and transcribed into RNA using T7 RNA polymerase. Subsequently, Biotin RNA Labeling Mix (Roche, Indianapolis, IN) was applied to biotinylate SNHG1 RNA sequences, which were then digested using RNase‐free DNase I, and purified using an RNeasy Mini Kit (Qiagen, Valencia, CA). Exactly 1 μg tagged RNA was heated to 95°C in RNA structure buffer (10 mmol/L Tris pH7, 0.1 mol/L KCl, 10 mmol/L MgCl_2_) for approximately 2 minutes, then transferred onto the ice for 30 minutes to facilitate formation of secondary RNA structures. Thereafter, 3 μg cells were lysed using radioimmunoprecipitation assay (RIPA) buffer (Sigma‐Aldrich, St Louis, MO) at 4°C for 1 hour. Lysates were centrifuged (12 000*g*, 4°C for 10 minutes) and supernatants were transferred into RNase‐free centrifugal tubes. Subsequently, 400 ng tagged RNA and 500 μL RNA immunoprecipitation (RIP) buffer was added to each tube before 1 hour incubation at room temperature. The lysate‐RNA mixture was washed using RIP buffer, and wash supernatant was subjected to RT‐PCR. After addition of 5× loading buffer and incubation at 95°C for 5 minutes, Western blot analysis was performed.

### 2.8 Western blot analysis

Protein expression of RICTOR (cat. No: #2114, Cell Signaling Technology, Danvers, MA), AKT (cat. no: sc‐135829; Santa Cruz Biotechnology), p‐AKT (cat. no. sc‐514032; Santa Cruz Biotechnology), SGK1 (cat. no: sc‐130402; Santa Cruz Biotechnology), p‐SGK1 (cat. no: sc‐398164; Santa Cruz Biotechnology), p70S6K1 (cat. no: sc‐393967; Santa Cruz Biotechnology), p‐p70S6K1 (cat. no: sc‐8416; Santa Cruz Biotechnology), LC3II/LC3I (cat. no: sc‐376404; Santa Cruz Biotechnology), and GADPH (cat. no: SAB2103104; Sigma‐Aldrich) (cat. no: sc‐293335; Santa Cruz Biotechnology) was determined by Western blot analysis. Protein quantities were estimated using a bicinchoninic acid (BCA) Protein Array Kit (Thermo Fisher Scientific, Rockford, IL). Proteins were then separated by molecular weight using SDS‐PAGE (8%‐15% polyacrylamide) and blotted onto a polyvinylidene difluoride (PVDF) membrane (Bio‐Rad Laboratories, Hercules, CA). Membranes were incubated with blocking reagent for 1 hour, and then with primary antibodies at 4°C overnight. After washing membranes with phosphate‐buffered saline with Tween‐20 (PBST), they were incubated with specific secondary antibodies (cat. no: sc‐2357 and sc‐516102, Santa Cruz Biotechnology) for 1 hour at room temperature. Membranes were washed using PBST before visualization with enhanced chemiluminescence reagent (Thermo Fisher Scientific), according to the manufacturer's instructions.

### 2.9 Flow cytometry

Target plasmids or negative controls were transfected into cell lines in six‐well plates (as described above). After 48 hours, cells were incubated with propidium iodide (PI; Sigma‐Aldrich) in the dark for 30 minutes, then harvested and subjected to flow cytometry. Cell cycling is expressed as the percentage of cells in G0/G1, S, and G2/M.

### 2.10 Transwell migration assay

At 48 hours posttransfection, approximately 2 × 10^4^ transfected CRC cells were resuspended in serum‐free medium and inoculated into Matrigel‐coated upper chambers of transwell cell migration assay plates (Corning Incorporated, Corning, NY) and complete DMEM with 10% FBS was added into the bottom chambers as a chemoattractant. After 24 hours of incubation at 37°C in 5% CO_2_, upper chamber cells were scraped. Subsequently, cells adherent to the lower chamber surface were stained with crystal violet for 2 hours before microscopy‐based imaging and counting.

### 2.11 Cell proliferation detection

Cell viability was evaluated by using Cell Counting Kit‐8 (CCK‐8, Beyotime Institute of Biotechnology, Shanghai, China). LoVo cells (5 × 10^3^ cells per well) were seeded into 96‐well plates and incubated overnight. Then, cells were transfected with siRICTOR2 or/and miR‐137 inhibitor for 48 hours. Later on, 10 μL CCK‐8 reagent was added into each well for another 2 hours. The absorbance (at 450 nm) of each well was detected with a microplate reader.

Next, cells were permeabilized with by 1% Triton X‐100 for 10 minutes and blocked with 4% bovine serum albumin (BSA) in PBS for 1 hour. Then, cells were incubated with primary anti‐Ki67 (Abcam Cambridge, MA, USA) for 1 hour at room temperature. Finally, cells were stained incubated with 4′,6‐diamidino‐2‐phenylindole (DAPI) (Sigma‐Aldrich) for 15 minutes. The images were obtained with a fluorescent microscope. Ki67‐postive cell rate was counted in three fields (×200 magnification).

### 2.12 MDC staining

LoVo cells were transfected with siR‐SNHG1, miR‐137 mimics, or miR‐137 inhibitor for 48 hours. After that, the cells were incubated with 50 μM monodansylcadaverine (MDC) at 37°C for 10 minutes in the dark. Later on, cells were washed with PBS for three times, and images were obtained with a fluorescent microscope.

### 2.13 Animal study

To establish an in vivo metastatic cancer model, 1 × 10^6^ cells transfected with siRNA–control (si‐NC), si‐SNHG1, miR‐137 inhibitor, or si‐SNHG1 + miR‐137 inhibitor were subcutaneously inoculated on the posterior flank of 6‐week‐old female nude mice (four mice per group). After injection, tumor size was measured weekly. Tumor volume was calculated according to the equation: volume = 0.5 × width × width × length. Mice were killed 4 weeks after inoculation. During the experiment, all animals were maintained under specific pathogen‐free conditions conforming to the relevant guidelines and regulations for the care and use of laboratory animals; the experimental protocol was approved by the Institutional Animal Care and Use Committee at The First Affiliated Hospital of Zhengzhou University.

### 2.14 Immunohistochemistry

To evaluate the expression of RICOTR in tumor tissues, formalin‐fixed and paraffin‐embedded tissues were subjected to immunohistochemical staining using anti‐RICTOR antibodies. After deparaffinization and rehydration, tissue sections were incubated with 3% hydrogen peroxide, and endogenous peroxidase was quenched in methanol. Tissue sections were blocked with 1% bovine serum albumin for 30 minutes before incubation with antibodies at 4℃ overnight. As a negative control, staining was also performed in the absence of primary antibodies for each tissue section. Tissue sections were then washed with PBS and incubated with secondary antibodies (Santa Cruz) for 1 hour. The enzyme reaction product was visualized using a diaminobenzidine staining kit (TIANGEN Biotech, Beijing, China), including counterstaining with hematoxylin.

### 2.15 Statistical analysis

Analysis was performed with SPSS Statistical software, version 19.0 (IBM, Chicago, IL). A paired‐samples *t* test was conducted to compare expression levels between CRC and adjacent normal tissues. The *χ* ^2^ test was used to investigate correlations between SNHG1 expression and clinicopathological features. An independent‐samples t‐test was conducted to compare values of gene expression, S phase fraction, number of migratory cells between different groups. Kaplan‐Meier analysis was conducted to analyze the association between SNHG1 expression and survival. *P* < .05 was considered as statistical difference.

## 3 RESULTS

### 3.1 SNHG1 expression is upregulated in CRC

**Figure 1:**
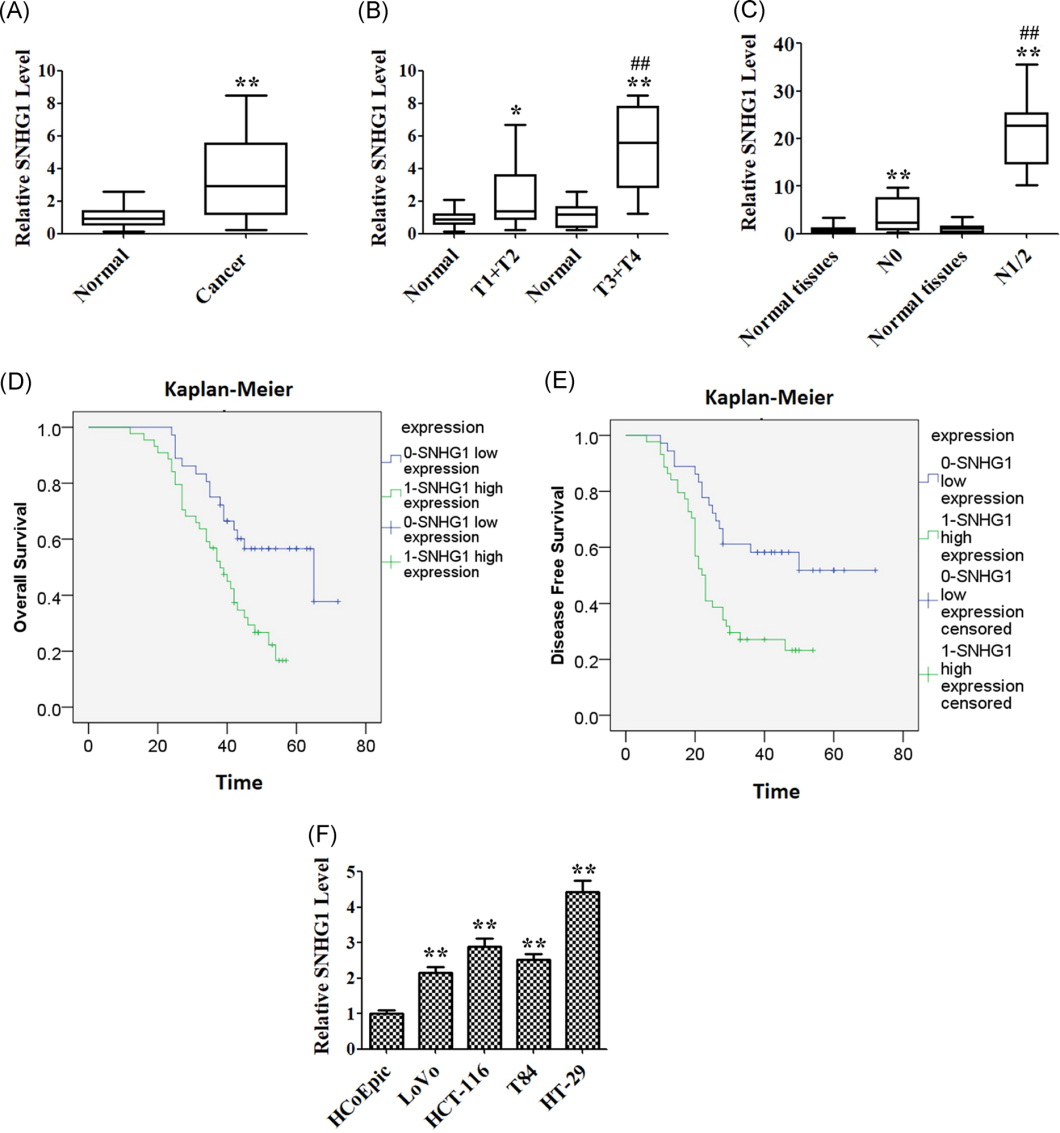
Clinical significance of SNHG1 level in colorectal cancer (CRC) cells. (A) The level of SNHG1 was detected using quantitative real‐time polymerase chain reaction (qRT‐PCR) assay in CRC tissues and normal tissues (n = 40). (B) qRT‐PCR assay was used to detect the level of SNHG1 in tumor tissues of different TNM stages (T1 + T2 or T3 + T4) and normal tissues, respectively. (C) QRT‐PCR assay was used to detect the level of SNHG1 in tumor tissues from patients with (N1 + N2) or without (N0) lymphatic metastasis, respectively. (D) Kaplan‐Meier analysis revealed that high expression of SNHG1 was related to the poor overall survival of CRC patients. (E) Kaplan‐Meier analysis revealed that high expression of SNHG1 was related to poor disease‐free survival of CRC patients. (F) The expression of SNHG1 in four CRC cell lines (LoVo, HT‐29, T84, HCT116) and a nontumor colorectal epithelial line (HCoEpic) were detected using qRT‐PCR assay. Data represent mean ± *SD*, n = 3; *P < .05, **P < .01 compared with the normal or HCpEpic group; ^##^ *P* < .01 compared with T1 + T2 or N0 group [Color figure can be viewed at wileyonlinelibrary.com]

Relative SNHG1 expression level in 80 pairs of CRC tissues and corresponding normal tissues was examined by qRT‐PCR. Compared with the normal group, SNHG1 expression was significantly upregulated in CRC tissues group (Figure 1A). As indicated in Figure 1B, the expression of SNHG1 was higher in patients with CRC in T3 and T4, compared with that in T1 and T2. In addition, the level of SNHG1 was markedly upregulated in patients with lymph node metastasis (N1/2) group, compared with patients without lymph node metastasis (N0) group (Figure 1C). Detailed correlations between SNHG1 expression and clinicopathological features are summarized in Table 1. As mentioned, upregulated SNHG1 expression was significantly positively correlated with lymph node metastasis (*P* = .033) and advanced TNM stage (*P* = .01). However, no significant associations were noted between SNHG1 and sex, age, location, tumor size, or tumor differentiation. Moreover, Kaplan‐Meier analysis demonstrated that SNHG1 high expression was positively associated with overall survival and disease free survival (Figure 1D and 1E). Taken together, these data indicated that upregulated SNHG1 expression was closely associated with the progression of CRC.

**Table 1:** The correlation between SNHG1 expression and clinicopathological features

Characteristics	Group	Number	IncRNA SNHG1		*P* value
			Low	High	
Sex	Male	47	20	27	.6
	Female	33	16	17	
Age, y	<60	38	18	20	.685
	≥60	42	18	24	
Tumors, cm	<5	33	14	19	.698
	≥5	47	22	25	
Location	Colon	35	15	20	.734
	Rectum	45	21	24	
Differentiation	WD	41	19	22	.805
	MD + PD	39	17	22	
TNM stage	I‐II	32	20	12	.01*
	III‐IV	48	16	32	
Lymph node metastasis	Yes	46	16	30	.033*
	No	34	20	14	

QRT‐PCR was used to detect the expression of SNHG1 in HCoEpic cell line and CRC LoVo, HCT‐116, T84, and HT‐29 cell lines. The level of SNHG1 was significantly increased in four CRC cells, compared with that of HCoEpic cells (Figure 1F). The highest expression of SNHG1 was observed in HT‐29 cells, while the lowest expression of SNHG1 was observed in LoVo cells. Therefore, HT‐29 and LoVo cells were chosen for subsequent functional studies.

### 3.2 miR‐137 is a biological target of SNHG1

**Figure 2:**
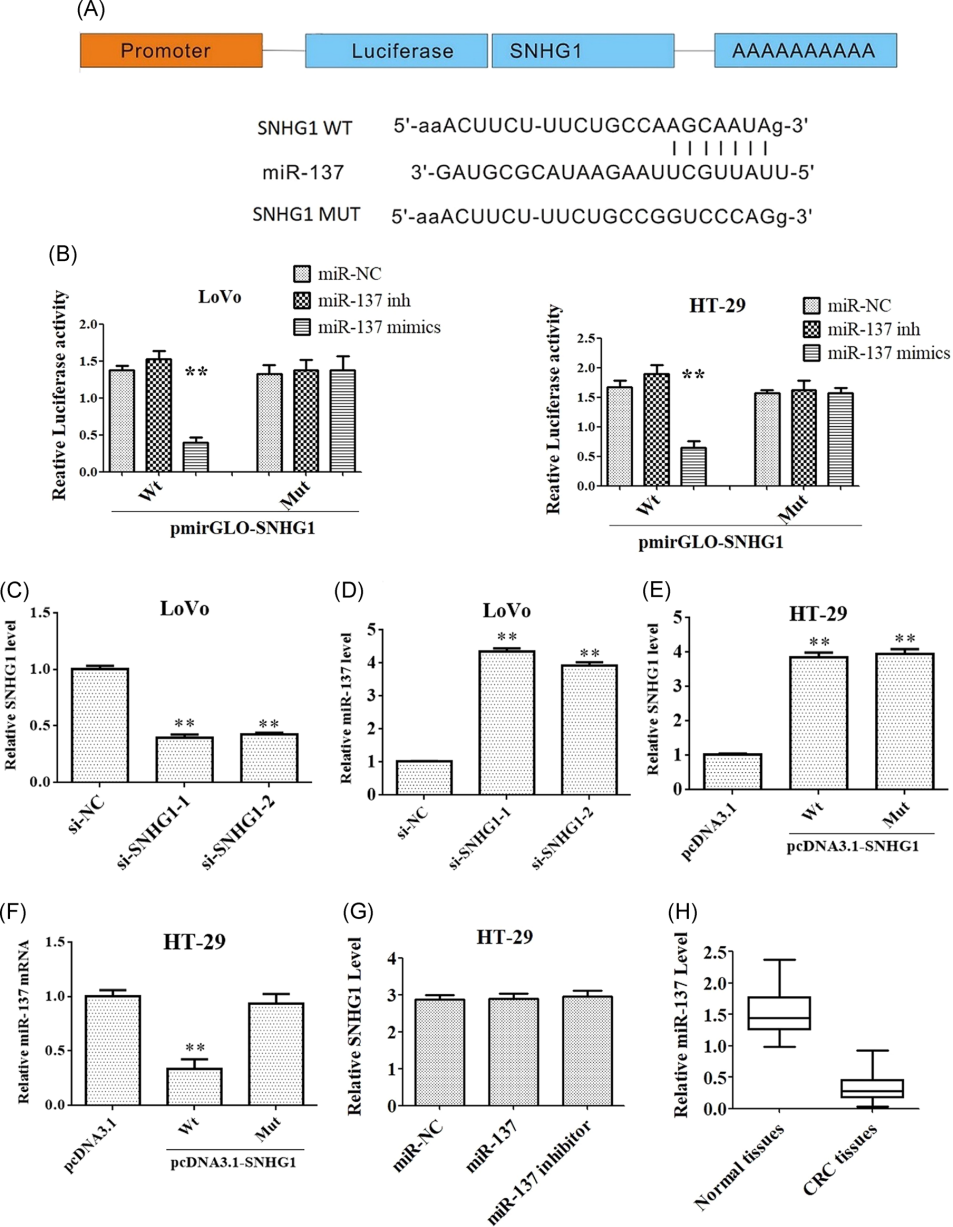
miR‐137 is identified as a biological target of SNHG1. (A) Predicted binding sites between SNHG1 and miR‐137. (B) SNHG1 Wt (or Mut) and corresponding plasmids (miR‐137 mimics or miR‐137 inhibitor) were cotransfected into LoVo and HT29 cells, respectively. The luciferase activity was measured by using the dual‐luciferase reporter assay. (C and D) LoVo cells were transfected with the si‐SNHG1 and si‐SNHG2 for 48 hours. The levels of SNHG1 and miR‐137 were detected via quantitative real‐time polymerase chain reaction (qRT‐PCR), respectively. (E and F) HT‐29 cells were transfected with SNHG1 Wt or SNHG1 Mut for 48 hours, respectively. The levels of SNHG1 and miR‐137 were detected via qRT‐PCR, respectively. (G) HT‐29 cells were transfected with miR‐137 mimics or inhibitor, respectively. The level of SNHG1 was detected via qRT‐PCR. (H) The level of miR‐137 was detected using qRT‐PCR assay in colorectal cancer tissues and normal tissues (n = 40). Data represent mean ± SD; **P < .01 compared with the control group. Mut, mutant; WT, wild‐type [Color figure can be viewed at wileyonlinelibrary.com]

Accumulating evidence indicates that lncRNAs act as “sponges” which regulate the expression and activity of miRNA. By exploring the bioinformatic database, Starbase, we found that miR‐137 was a hypothetic miRNA targeting SHNG1 (Figure 2A). To confirm whether miR‐137 is a direct binding target of SNHG1, a luciferase reporter gene assay was performed. As shown in Figure 2B and 2C, overexpression of miR‐137 significantly reduced SNHG1‐WT reporter activity in LoVo and HT‐29 cells, respectively. However, it failed to repress the mutated SNHG1‐3′UTR. To avoid off‐target effects, we designed two siRNAs targeting different SNHG1 regions. As indicated in Figure 2D, the level of SNHG1 was significantly decreased following transfection with si‐SNHG1s. In addition, downregulation of SHNG1 markedly increased the level of miR‐137 (Figure 2).

Ectopic SNHG1 overexpression (pcDNA3.1‐SNHG1) increased the transcription level of SNHG1 in HT‐29 cells (Figure 2E). In addition, the level of miR‐137 was notably decreased following transfection with pcDNA3.1‐SNHG1‐Wt (Figure 2F). Expression of SNHG1 was unaffected following transfection with miR‐137 (Figure 2G). Meanwhile, the expression level miR‐137 was significantly downregulated in CRC tissues, compared with that in normal tissues (Figure 2H). These results suggested that MiR‐137 is a biological target of SNHG1.

### 3.3 miR‐137 is negatively mediated by SNHG1

**Figure 3:**
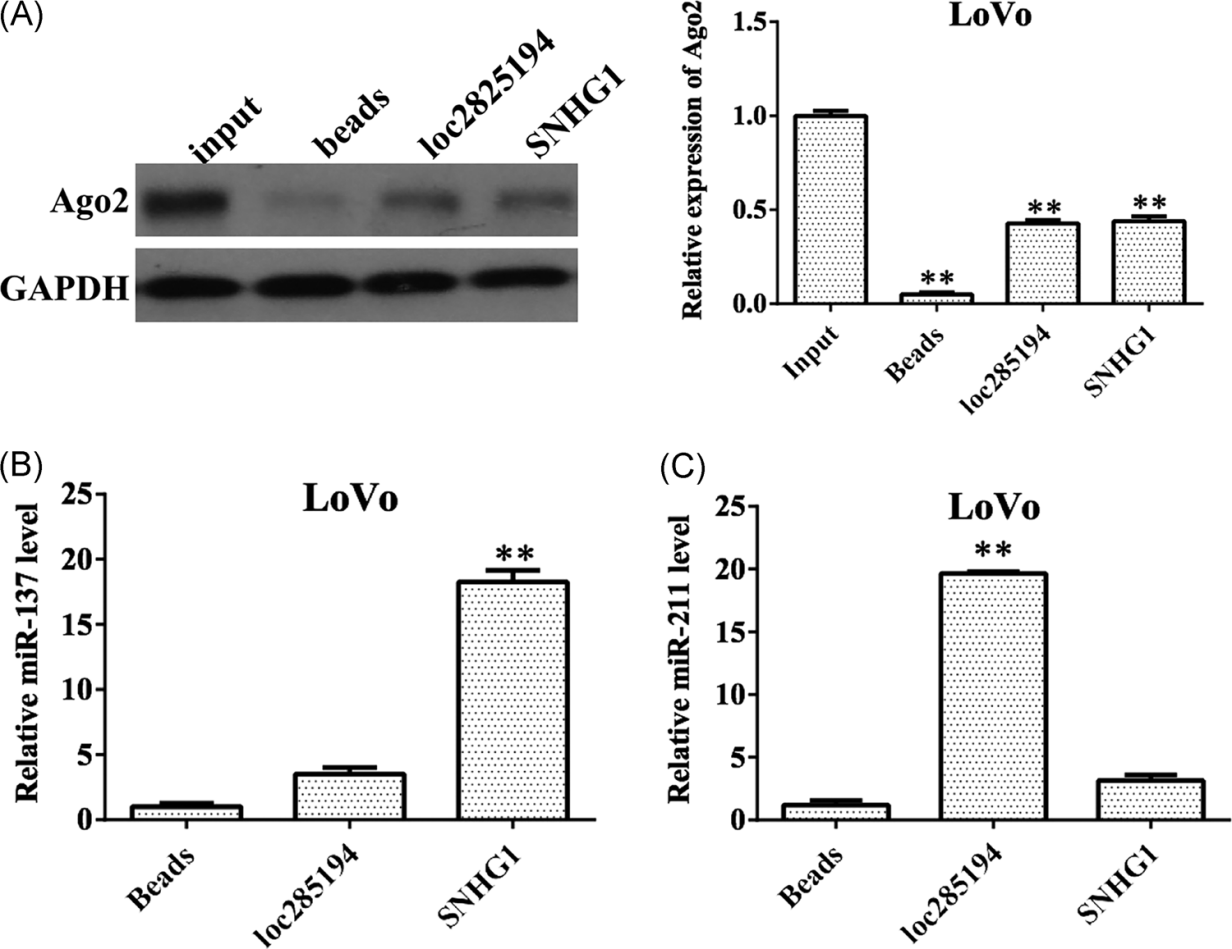
The activity of SNHG1 is mediated through negative regulation of miR‐137. (A) Identification of Ago2 and SNHG1 in the same RISC complex by RNA pull‐down assay. Pull‐down of Ago2 by biotin‐labeled SNHG1 or loc285194 RNA probe was detected by Western blot analysis. SNHG1 in the RNA‐precipitated samples was detected using quantitative real‐time polymerase chain reaction (qRT‐PCR). Empty vectors (magnetic beads) were used as a negative control, and loc285194 was used as the positive control. (B) Identification of miR‐137 and SNHG1 in the same RISC complex by RNA pull‐down assay. SNHG1 in the RNA‐precipitated samples was detected using qRT‐PCR. (C) Identification of miR‐211 and SNHG1 in the same RISC complex by RNA pull‐down assay. SNHG1 in the RNA‐precipitated samples was detected using qRT‐PCR. Data represent mean ± SD, n = 3; **P < .01 compared with the control group. GAPDH, glyceraldehyde‐3‐phosphate dehydrogenase

Previous studies have demonstrated that miRNAs exist in the cytoplasm in the form of miR‐nucleoprotein complexes, including the protein Ago2, which is a key RISC component with an important role in siRNA or miR‐induced gene silencing.^20^ Ago2 coimmunoprecipitation may thus facilitate identification of potential miRNA targets. An RNA pull‐down assay was carried out using SNHG1 probes. Meanwhile, Ago2 and miR‐137 were investigated to determine whether SNHG1 and miR‐137 exist in the same RISC complex. RNA pull‐down assays were performed to determine the physical relationship between SNHG1 and Ago2 (Figure 3A). To confirm that miR‐137 and SNHG1 are in the same Ago2 complex, we synthesized biotin‐labeled SNHG1 RNA probe and mixed with the cellular extract. After pull‐down experiment with beads, we detected miR‐137 by Western blot analysis, suggesting SNHG1 directly interacted with miR‐137 (Figure 3B). To further investigate the relationship between SNHG1 and miR‐137, we examined miR‐211, which was negatively regulated by loc285194. The qRT‐PCR assay results demonstrated that a significant amount of miRNA‐211 in the loc285194 pulled down pellet, while the amount of miR‐211 in the SNHG1 pulled down pellet was only slightly increased compared with control (Figure 3C). These data indicated that the activity of SNHG1 is mediated through negative regulation of miR‐137.

### 3.4 RICTOR is a target gene of miR‐137

**Figure 4:**
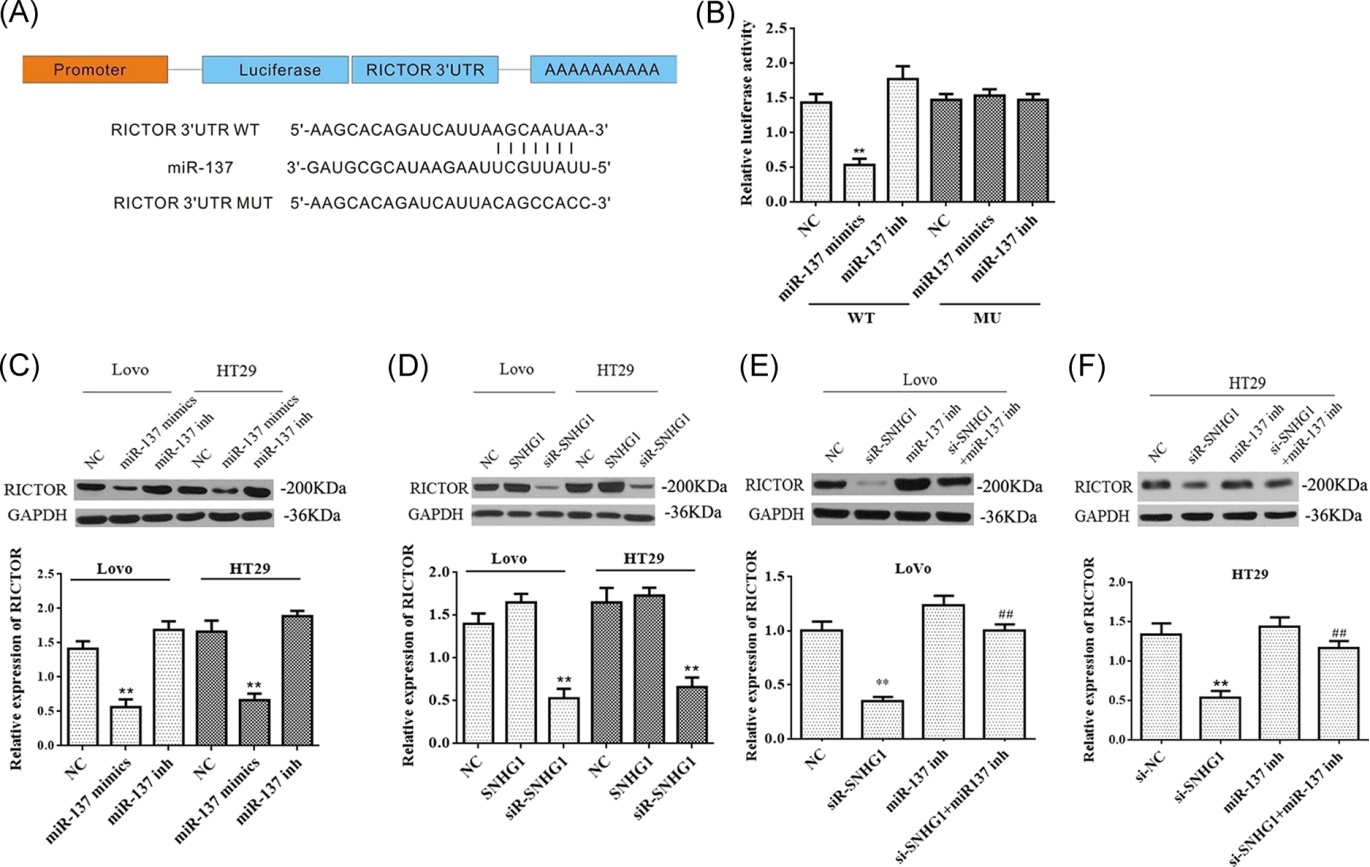
RICTOR is a target gene of miR‐137 and is regulated by SNHG1. (A) Predicted binding sites between miR‐137and RICTOR. (B) RICTOR Wt (or Mut) and corresponding plasmids (miR‐137 mimics or miR‐137 inhibitor) were cotransfected into LoVo cells, respectively. The luciferase activity was measured by using the dual‐luciferase reporter assay. (C) LoVo and HT‐29 cells were transfected with miR‐137 mimics or inhibitor, respectively. The level of RICTOR was detected via Western blot analysis. (D) LoVo and HT‐29 cells were transfected with SNHG1 mimics or siSNHG1, respectively. The level of RICTOR was detected via Western blot analysis. (E and F) LoVo and HT‐29 cells were transfected with siSNHG1, miR‐137 inhibitor or si‐SNHG1 + miR‐137 inhibitor, respectively. The level of RICTOR was detected via Western blot analysis. Data represent mean ± SD, n = 3; **P < .01 compared with the control group; ^##^ *P* < .01 compared with si‐SNHG1 group. NC, negative control; Mut, mutant; WT, wild‐type [Color figure can be viewed at wileyonlinelibrary.com]

TargetScan, PITA, and miRanda software were used to predict the downstream targets of miR‐137. The results showed that RICTOR is a potential target of miR‐137 (Figure 4A). To validate whether RICTOR is a downstream target of miR‐137, luciferase reporter plasmids containing the RICTOR miR‐137‐binding sites (WT), or a mutant RICTOR 3′UTR were constructed. As indicated in Figure 4B, overexpression of miR‐137 significantly reduced luciferase activity of RICTOR‐WT but not the activity of the RICTOR‐MUT in HEK293T cells, demonstrating that miR‐137 could specifically target the RICTOR 3'UTR. In addition, Western blot analysis was used to confirm the results. Upregulation of miR‐137 markedly decreased the level of RICTOR in LoVo and HT‐29 cells, while downregulation of miR‐137 had no effect on RICTOR levels (Figure 4C).

To confirm whether SNHG1 regulates RICTOR expression in LoVo and HT‐29 cells, we firstly analyze the expression of RICTOR following transfection with siR‐SNHG1. As shown in Figure 4D, the downregulation of SNHG1 markedly decreased the level of RICTOR. However, the level of RICTOR was significantly increased after cotransfection with si‐SNHG1 and miR‐137 inhibitor in LoVo and HT‐29 cells, respectively (Figure 4E and 4F). These data suggest that RICTOR is a direct target of miR‐137, and that SNHG1 can regulate RICTOR expression through interaction with miR‐137.

### 3.5 SNHG1 exerts carcinogenic activity through regulation of the mTORC2 pathway in vitro

**Figure 5:**
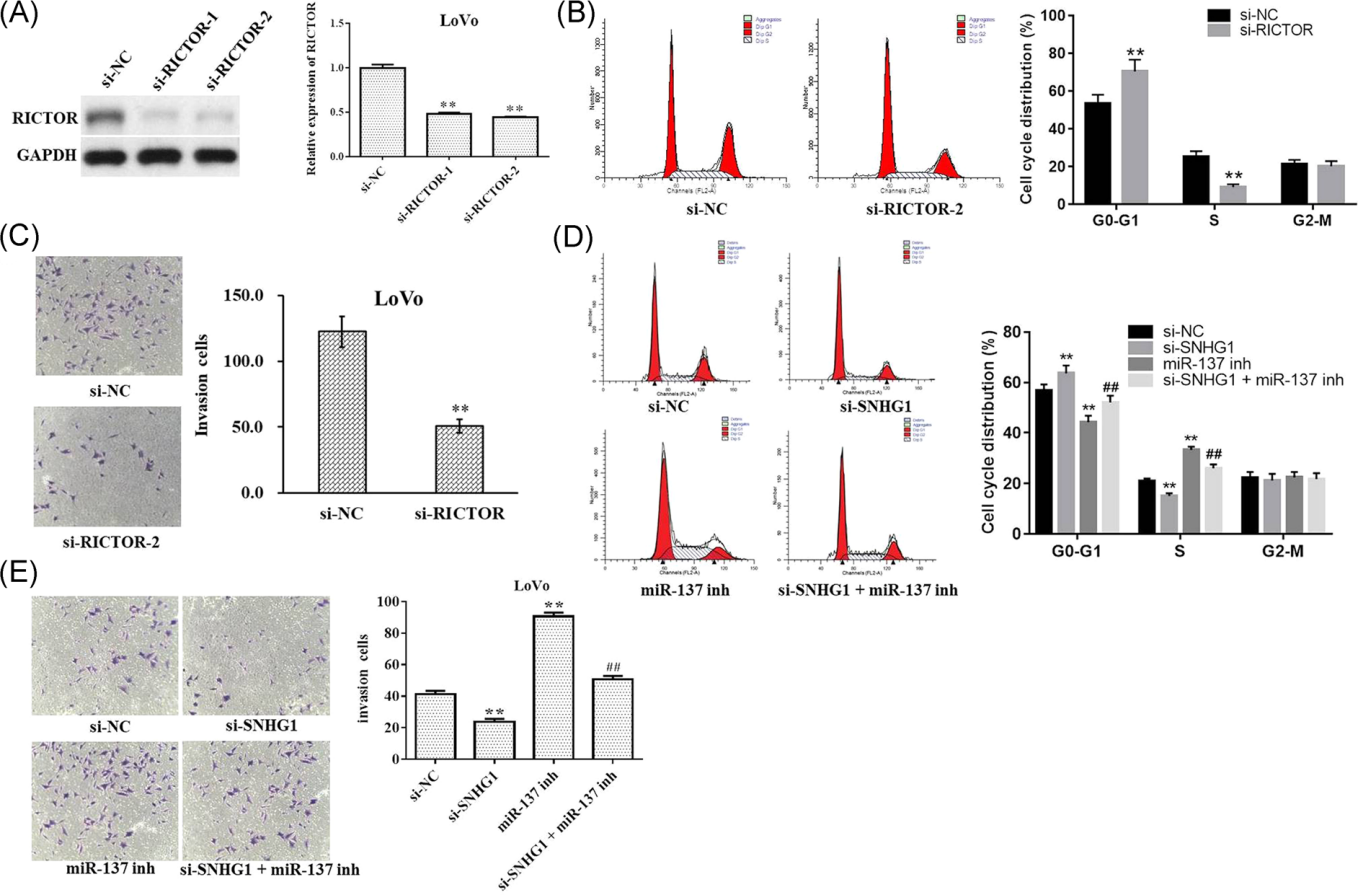
Carcinogenic activity of SNHG1 is mediated through regulation of RICTOR in vitro. (A) LoVo cells were transfected with si‐RICTOR‐1 or si‐RICTOR‐2 for 48 hours, respectively. The level of RICTOR was detected via Western blot analysis. (B) LoVo cells were transfected with si‐RICTOR‐2 for 48 hours. Propidium iodide (PI) and flow cytometry was used to analyze the cell‐cycle distribution. (C) Invasion assays were performed with transfected cells using Transwell inserts. (D) LoVo cells were transfected with siSNHG1, miR‐137 inhibitor or si‐SNHG1 + miR‐137 inhibitor, respectively. PI and flow cytometry were used to analyze the cell cycle distribution. (E) Invasion assays were performed with transfected cells using Transwell inserts. Data represent mean ± SD, n = 3; **P < 0.01 compared with the control group; ^##^ *P* < 0.01 compared with si‐SNHG1 group. GAPDH, glyceraldehyde‐3‐phosphate dehydrogenase; NC, negative control [Color figure can be viewed at wileyonlinelibrary.com]

For further study, we investigated the effects of RICTOR on CRC cells. As shown in Figure 5A, the expression of RICTOR was decreased the most following transfection with si‐RICTOR‐2. In addition, the percentage of the S phase was markedly decreased in the si‐RICTOR‐2 group, compared with the si‐NC group (Figure 5B). Meanwhile, downregulation of RICTOR significantly inhibited the invasion ability of LoVo cell (Figure 5C).

**Figure 6:**
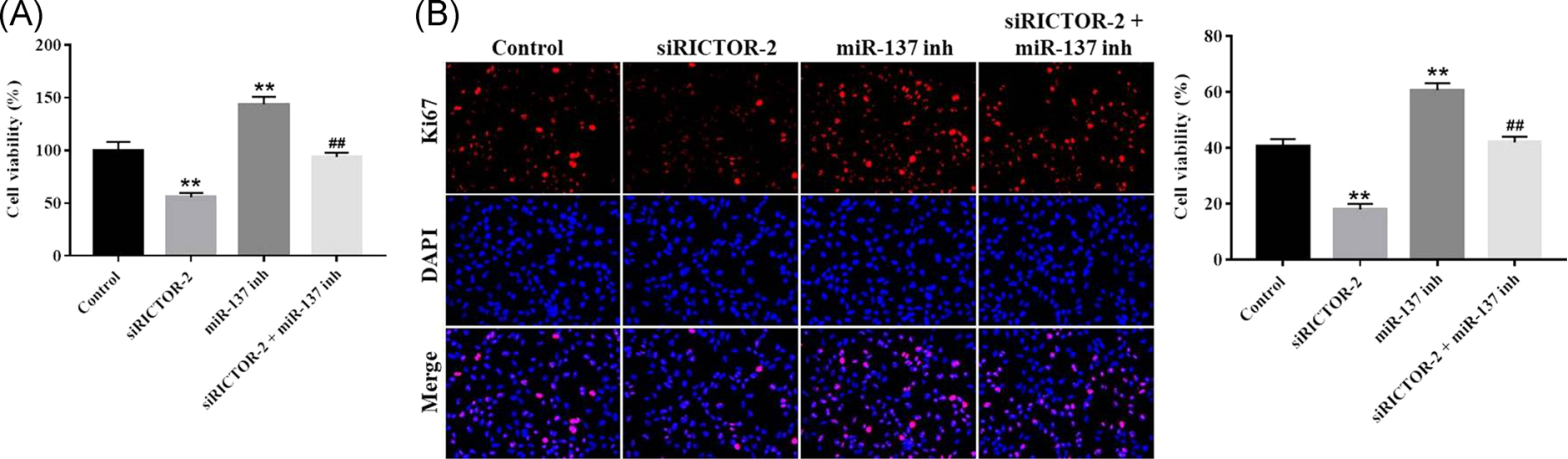
Knockdown of RICTOR restored the effect of miR‐137 inhibitor on cell proliferation. (A) LoVo cells were transfected with si‐RICTOR‐2, miR‐137 inhibitor or si‐RICTOR‐2 + miR‐137 inhibitor for 48 hours, respectively. Cell Counting Kit 8 (CCK8) assay was used to evaluate cell proliferation. (B) LoVo cells were incubated with Ki67 or 4′,6‐diamidino‐2‐phenylindole (DAPI) for detection of cell proliferation. Data represent mean ± SD, n = 3; **P < .01 compared with the control group; ^##^ *P* < .01 compared with miR‐137 inhibitor group [Color figure can be viewed at wileyonlinelibrary.com]

As shown in Figure 5D, the downregulation of SNHG1 significantly inhibited the percentage of the S phase. However, downregulation of miR‐137 notably increased the percentage of the S phase, which were markedly reversed after cotransfection with si‐SNHG1 (Figure 5D). In addition, the invasion ability of LoVo cell was significantly increased after transfection with miR‐137 inhibitor, while the pro‐invasive effect was notably decreased after cotransfection with si‐SNHG1 (Figure 5E). Moreover, cell proliferation study indicated miR‐137 inhibitor notable increased cell proliferation, while there effects were completely blocked by si‐RICTOR2 (Figure 6A and 6B). All these data suggest that RICTOR may influence CRC cell proliferative and invasive capabilities.

**Figure 7:**
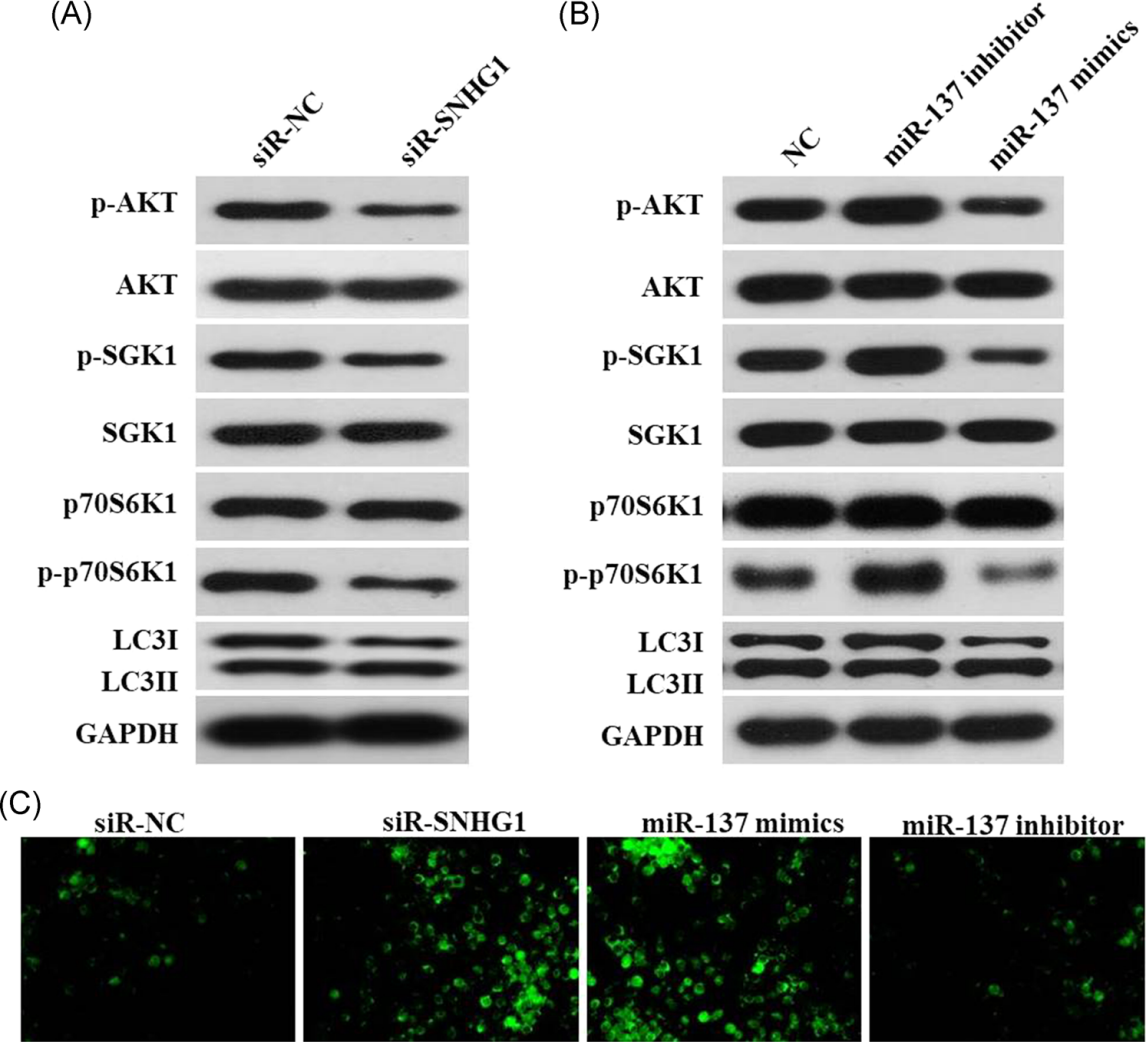
Carcinogenic activity of SNHG1 was mediated through regulation of the mTORC2 pathway in vitro. (A) LoVo cells were transfected with si‐SNHG1 for 48 hours. P‐AKT, AKT, p‐SGK1, SGK1, p70S6K1, p‐p70S6K1, LC3 I, and LC3 II were detected in LoVo cells after transfection for 48 hours. (B) LoVo cells were transfected with miR‐137 inhibitor or miR‐137 mimics for 48 hours, respectively. P‐AKT, AKT, p‐SGK1, SGK1, p70S6K1, p‐p70S6K1, LC3 I, and LC3 II were detected in LoVo cells after transfection for 48 hours. (C) LoVo cells were subjected to MDA staining. GAPDH, glyceraldehyde‐3‐phosphate dehydrogenase [Color figure can be viewed at wileyonlinelibrary.com]

To further validate that SNHG1 and miR‐137 exert biological activities mediated through the regulation of RICTOR, the expressions of RICTOR‐associated proteins were detected. As shown in Figure 7A and 7B, downregulation of SNHG1 or upregulation of miR‐137 significantly decreased the expressions of phosphorylation of AKT, SGK1, and p70S6K1 and increased the level of L3II/LC3I, respectively. In contrast, downregulation of miR‐137 significantly increased the expressions of phosphorylation of AKT, SGK1, and p70S6K1 and decreased the level of L3II/LC3I. In addition, downregulation of SNHG1 or upregulation of miR‐137 notably increased MDA‐positive cells, while downregulation of miR‐137 slightly decreased MDA‐positive cells (Figure 7C).

### 3.6 SNHG1 exerts carcinogenic activity in vivo through regulation of miR‐137

**Figure 8:**
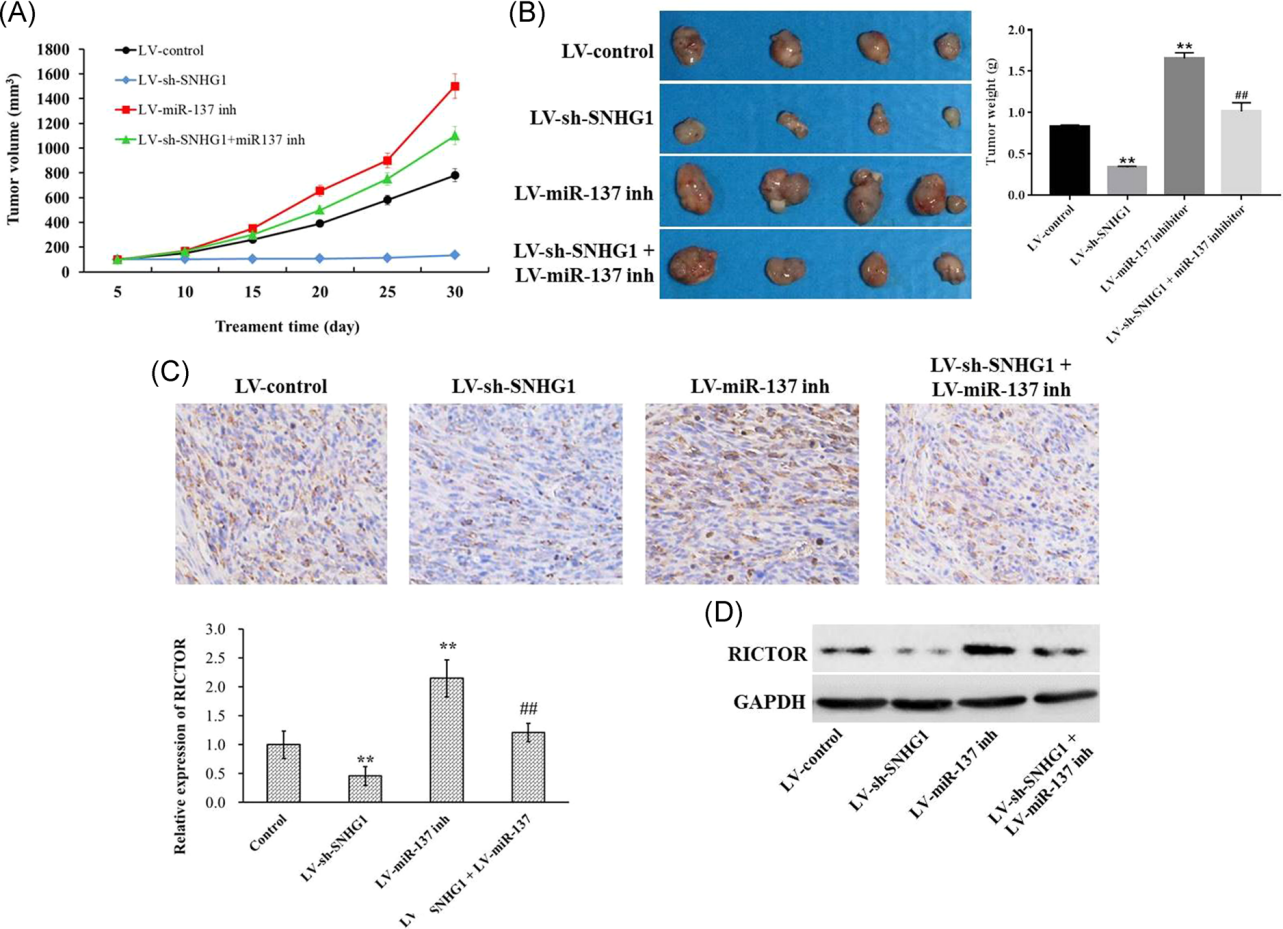
Downregulation of SNHG1 inhibited tumor growth in vivo. (A) A total of 1 × 10^6^ cells transfected with siRNA–control (si‐NC), si‐SNHG1, miR‐137 inhibitor, or si‐SNHG1 + miR‐137 inhibitor were subcutaneously inoculated on the posterior flank of 6‐week‐old female nude mice. The tumor volumes were monitored weekly. (B) The tumors were isolated from mice and weighted in 4 weeks. (C and D) The expression of RICTOR in tumor tissues was detected with immunohistochemistry staining and Western blot analysis. Data represent mean ± SD, n = 3; **P < .01 compared with control group [Color figure can be viewed at wileyonlinelibrary.com]

To further demonstrate that the carcinogenic activity of SNHG1 is mediated through negative regulation of miR‐137, LoVo cells were subcutaneously injected into nude mice. Four weeks later, tumor volume and tumor weight were significantly decreased in the LoVo‐si‐SNHG1 group (Figure 8A and 8B). Downregulation of miR‐137 markedly increased the tumor weight and volume, which were significantly decreased following transfection with sh‐SNHG1 (Figure 8A and 8B). Additionally, immunohistochemistry (IHC) results indicated that the level of RICTOR was markedly decreased after transfection with sh‐SNHG1, which was significantly increased after transfection with miR‐137 inhibitor. However, the upregulated RICTOR level in miR‐137 inhibitor group was notably downregulated following cotransfection with sh‐SNHG1 (Figure 8C and 8D). These data indicate that SNHG1 exerts carcinogenic activity in vivo through regulation of miR‐137.

## 4 DISCUSSION

It is known that lncRNAs play functional roles in a series of steps during tumor development, and they may interact with DNA, RNA, protein and/or various combinations to act as important regulators of chromatin organization and transcriptional as well as posttranscriptional regulation.^21^ Recent studies indicate that the ncRNA SNHG1 plays important roles in tumor development, and have provided new insights into the biological activities of SNHG1 in tumors (including CRC).^22^ However, the roles of lncRNAs in CRC tumorigenesis remain incompletely understood. The current study demonstrates that the oncogenic activity of SNHG1 in CRC is mediated through regulation of the miR‐137/RICTOR axis.

Firstly, we found that SNHG1 expression was upregulated in CRC compared with adjacent normal tissues. Sun et al^23^ similarly report that SNHG1 expression is upregulated in stage 3/4 tumors and metastasis (although these terms are not well defined in their study). The current study found that SNHG1 expression positively correlates with advanced TNM stage (III and IV) and the presence of lymph node metastasis. In addition, Kaplan‐Meier analysis demonstrated a correlation between high tumor SNHG1 expression and reduced survival (including OS and DFS interval), suggesting that SNHG1 may be useful as a diagnostic and prognostic indicator in CRC patients. Though such preliminary results indicate the clinical significance of SNHG1 in CRC, underlying mechanisms remain unclear.

Accumulating recent evidence indicates that certain lncRNAs share common miR‐binding sites with miRNA target genes, suggesting a mechanism by which lncRNAs may regulate miR‐mediated target repression. Abnormal expression of such lncRNA/miRNA‐mRNA pairs in specific tissues or organs confers the capacities for tumorigenesis, progression, and metastasis.^24^, ^25^, ^26^, ^27^ A recent study showed that SNHG1 promotes cell proliferation in CRC by sequestering miR‐145,^28^ which indicates that SNHG1/miRNA interactions may play important roles in CRC.

The current study used Starbase v2.0 online software to identify 27 miRNAs exhibiting bp complementarity with SNHG1. These included a subset of miRNAs already previously identified as sponges of SNHG1, (eg, miR‐145, miR‐195, and miR‐101‐3p), supporting reliability of prediction software results. Expression changes of these 27 miRNAs were measured after SNHG1 silencing, and expression of miR‐137 was found to be the most upregulated, while ectopic SNHG1 expression lowered miR‐137 expression in both LoVo and HT‐29 cells. Luciferase reporter gene assays demonstrated that miR‐137 directly binds SNHG1. It has previously been documented that SNHG1 promoted cell proliferation by sequestering miR‐145 in CRC^28^; therefore, the current study also examined the expression of miR‐145 in LoVo and HT‐29 cells after SNHG1 silencing. We found that miR‐145 expression was significantly increased in LoVo but not HT‐29 cells, leading to miR‐137 being chosen for subsequent studies.

In addition, the biological activities of SNHG1 and miR‐137 in CRC cells were investigated. Results demonstrate that SNHG1 silencing inhibits proliferation and migration of CRC cells, and that miR‐137 inhibitor abrogates this inhibition, indicating that SNHG1 oncogenic activities, both in vivo and in vitro, are mediated through regulation of miR‐137 expression.

miR‐137 is located on human chromosome 1p22 and is involved in many biological processes and diseases.^29^ To date, research indicates that miR‐137 may play a dual role during tumorigenesis, and that the nature of this role may be dependent on tumor type and target messenger RNA (mRNA) identities.^30^, ^31^ As a tumor suppressor in melanoma, miR‐137 may inhibit cell migration by targeting the TBX3 transcription factor.^32^ As an oncogene in breast cancer, miR‐137 may enhance the epithelial‐to‐mesenchymal transition (EMT) capacity of tumor cells by inhibiting the function of BMP7.^33^ The current study demonstrated that miR‐137 is downregulated in CRC tissues and that its mimic may abrogate the oncogenic activities of SNHG1, suggesting that miR‐137 has a tumor suppressor role in CRC.

The expression of miR‐137 is frequently downregulated during oncogenesis, but its regulation and possible mechanisms remain to be clarified. It has been documented that methyl‐CpG‐binding protein 2 (MeCP2) and DNA methyltransferases (DNMTs) may work cooperatively to enhance methylation of the miR‐137 promoter, leading to transcriptional silencing.^34^ The current study found that SNHG1 may—instead of inhibiting expression of miR‐137—sequester mature miR‐137 (a novel posttranscriptional mechanism of miR‐137 regulation).

It is well known that lncRNAs may regulate other, usually protein‐coding, RNA transcripts by competing for common miRNAs. The current study found that SNHG1 and RICTOR share a common binding site for miR‐137, and that the transcription levels of SNHG1 and RICTOR in CRC. RICTOR is a subunit of mTORC2, and acts mainly as a regulator of AGC kinase phosphorylation/activation (including AKTSer473).^35^ Functionally, mTORC2 regulates cell growth and survival in response to many signals, such as hormones, cytokines, and pharmaceutical compounds. Overexpression of RICTOR has already been observed in several cancers, including colorectal, prostate, hepatocellular, and pancreatic.^36^, ^37^, ^38^, ^39^ In addition, two independent studies have demonstrated that RICTOR overexpression may indicate poorer prognosis.^37^, ^39^ Future studies may prove that combined RICTOR and SNHG1 expression may facilitate more precise prognostication in CRC.^40^

It was recently reported that RICTOR overexpression may cooperate with NRAS mutation to stimulate proliferation in melanomas.^41^ Furthermore, Gulhati et al^42^ demonstrated that the mTORC1/2 signaling pathway was involved in regulating EMT and metastasis in CRC. The current study examined events downstream of RICTOR after manipulating SNHG1 and miR‐137 expression. Results demonstrate that RICTOR silencing may inhibit CRC cell proliferation and invasion. In addition, si‐SNHG1 and miR‐137 mimic appears to decrease phosphorylation of AKT, SGK1, and p70S6K1, as well as increasing the LC3II/ LC3I ratio. In context, p‐AKT, p‐SGK1, and p‐p70S6K1 are known to promote proliferation, while an increased LC3II/I ratio may suggest activation of autophagy.^41^, ^42^ It is therefore reasonable to infer that SNHG1, miR‐137, and RICTOR may be involved in the regulation of proliferation and autophagy in CRC cells. Proliferation was inhibited while autophagy may be activated to promote the cells survive. The current data demonstrate a novel mechanism of regulating RICTOR expression at the posttranscriptional level, and this may inform the development of novel CRC treatment strategies targeting mTORC2.

## 5 CONCLUSION

The current study identifies a novel mechanism of lncRNA and miRNA interaction which appears to promote CRC tumorigenesis and progression. However, miR‐137 is not the only miRNA bound by SNHG1, as 27 miRNAs were identified that are expected to exhibit sufficient basepair complementarity for this purpose. Additionally, miR‐137 biological function is not RICTOR‐specific, as it is also able to bind the 3′UTR of mRNA for other protein‐coding genes, such as TBX3 and BMP7. Taken together, SNHG1 and miR‐137 likely engage in significantly more comprehensive biological functions than those observed in the present study. Future studies should identify additional oncogenic activities of SNHG1 in clinical samples from a larger number of patients. Such information will contribute to the development of novel targeted anticancer therapies.

## CONFLICT OF INTERESTS

The authors declare that there are no conflict of interests.

## DATA ACCESSIBILITY

The data that support the findings of this study are available from the corresponding author upon reasonable request.

## References

[1] Torre LA , Bray F , Siegel RL , Ferlay J , Lortet‐Tieulent J , Jemal A . Global cancer statistics, 2012. CA Cancer J Clin. 2015;65:87‐108. 25651787 10.3322/caac.21262

[2] Szaryńska M , Olejniczak A , Kmieć Z . The role of cancer stem cells in pathogenesis of colorectal cancer. Postępy Higieny i Medycyny Doświadczalnej. 2016;70:1469‐1482. 28100854 10.5604/17322693.1227897

[3] Brosens LAA , Offerhaus GJA , Giardiello FM . Hereditary colorectal cancer. Surg Clin North Am. 2015;95:1067‐1080. 26315524 10.1016/j.suc.2015.05.004 PMC4555838

[4] Dong X , Chen K , Cuevas‐Diaz Duran R , et al. Comprehensive identification of long non‐coding RNAs in purified cell types from the brain reveals functional LncRNA in OPC fate determination. PLOS Genet. 2015;11:e1005669. 26683846 10.1371/journal.pgen.1005669 PMC4980008

[5] Salmena L , Poliseno L , Tay Y , Kats L , Pandolfi PP . A ceRNA hypothesis: the Rosetta Stone of a hidden RNA language? Cell. 2011;146:353‐358. 21802130 10.1016/j.cell.2011.07.014 PMC3235919

[6] Thomson DW , Dinger ME . Endogenous microRNA sponges: evidence and controversy. Nat Rev Genet. 2016;17:272‐283. 27040487 10.1038/nrg.2016.20

[7] Karreth FA , Pandolfi PP . ceRNA cross‐talk in cancer: when ce‐bling rivalries go awry. Cancer Discov. 2013;3:1113‐1121. 24072616 10.1158/2159-8290.CD-13-0202 PMC3801300

[8] Tay Y , Rinn J , Pandolfi PP . The multilayered complexity of ceRNA crosstalk and competition. Nature. 2014;505:344‐352. 24429633 10.1038/nature12986 PMC4113481

[9] Wang Y , Chen F , Zhao M , et al. The long noncoding RNA HULC promotes liver cancer by increasing the expression of the HMGA2 oncogene via sequestration of the microRNA‐186. J Biol Chem. 2017;292:15395‐15407. 28765279 10.1074/jbc.M117.783738 PMC5602398

[10] Yu B , Ye X , Du Q , Zhu B , Zhai Q . The long non‐coding RNA CRNDE promotes colorectal carcinoma progression by competitively binding miR‐217 with TCF7L2 and enhancing the Wnt/β‐catenin signaling pathway. Cell Physiol Biochem. 2017;41:2489‐2502. 28472810 10.1159/000475941

[11] Xu J , Zhang R , Zhao J . The novel long noncoding RNA TUSC7 inhibits proliferation by sponging miR‐211 in colorectal cancer. Cell Physiol Biochem. 2017;41:635‐644. 28214867 10.1159/000457938

[12] Cui Y , Zhang F , Zhu C , Geng L , Tian T , Liu H . Upregulated lncRNA SNHG1 contributes to progression of non‐small cell lung cancer through inhibition of miR‐101‐3p and activation of Wnt/β‐catenin signaling pathway. Oncotarget. 2017;8:17785‐17794. 28147312 10.18632/oncotarget.14854 PMC5392286

[13] You J , Zhou Q , Fang N , et al. Noncoding RNA small nucleolar RNA host gene 1 promote cell proliferation in nonsmall cell lung cancer. Indian J Cancer. 2014;51(suppl 3):99. 10.4103/0019-509X.154092 25818744

[14] Zhang M , Wang W , Li T , et al. Long noncoding RNA SNHG1 predicts a poor prognosis and promotes hepatocellular carcinoma tumorigenesis. Biomed Pharmacother. 2016;80:73‐79. 27133041 10.1016/j.biopha.2016.02.036

[15] Sahu D , Hsu CL , Lin CC , et al. Co‐expression analysis identifies long noncoding RNA *SNHG1* as a novel predictor for event‐free survival in neuroblastoma. Oncotarget. 2016;7:58022‐58037. 27517149 10.18632/oncotarget.11158 PMC5295409

[16] Yan Y , Fan Q , Wang L , Zhou Y , Li J , Zhou K . LncRNA Snhg1, a non‐degradable sponge for miR‐338, promotes expression of proto‐oncogene CST3 in primary esophageal cancer cells. Oncotarget. 2017;8:35750‐35760. 28423738 10.18632/oncotarget.16189 PMC5482614

[17] Zhang H , Zhou D , Ying M , et al. Expression of long non‐coding RNA (lncRNA) small nucleolar RNA host gene 1 (SNHG1) exacerbates hepatocellular carcinoma through suppressing miR‐195. Med Sci Monit. 2016;22:4820‐4829. 27932778 10.12659/MSM.898574 PMC5167104

[18] Edge SB , Compton CC . The American Joint Committee on Cancer: the 7th edition of the AJCC cancer staging manual and the future of TNM. Ann Surg Oncol. 2010;17:1471‐1474. 20180029 10.1245/s10434-010-0985-4

[19] Livak KJ , Schmittgen TD . Analysis of relative gene expression data using real‐time quantitative PCR and the 2−ΔΔCT method. Methods. 2001;25:402‐408. 11846609 10.1006/meth.2001.1262

[20] Yang JS , Lai EC . Dicer‐independent, Ago2‐mediated microRNA biogenesis in vertebrates. Cell Cycle. 2010;9:4455‐4460. 21088485 10.4161/cc.9.22.13958 PMC3048044

[21] Akhade VS , Pal D , Kanduri C . Advances in experimental medicine and biology. Adv Exp Med Biol. 2017;1008:47‐74. 28815536 10.1007/978-981-10-5203-3_2

[22] Zhu Y , Li B , Liu Z , et al. Up‐regulation of lncRNA SNHG1 indicates poor prognosis and promotes cell proliferation and metastasis of colorectal cancer by activation of the Wnt/β‐catenin signaling pathway. Oncotarget. 2017;8:111715‐111727. 29340086 10.18632/oncotarget.22903 PMC5762354

[23] Sun X , Wang Z , Yuan W . Down‐regulated long non‐coding RNA SNHG1 inhibits tumor genesis of colorectal carcinoma. Cancer Biomarkers. 2017;20:67‐73. 28759957 10.3233/CBM-170112

[24] Wang J , Liu X , Wu H , et al. CREB up‐regulates long non‐coding RNA, HULC expression through interaction with microRNA‐372 in liver cancer. Nucleic Acids Res. 2010;38:5366‐5383. 20423907 10.1093/nar/gkq285 PMC2938198

[25] Kallen AN , Zhou XB , Xu J , et al. The imprinted H19 lncRNA antagonizes let‐7 microRNAs. Mol Cell. 2013;52:101‐112. 24055342 10.1016/j.molcel.2013.08.027 PMC3843377

[26] Han Y , Liu Y , Zhang H , et al. Hsa‐miR‐125b suppresses bladder cancer development by down‐regulating oncogene SIRT7 and oncogenic long noncoding RNA MALAT1. FEBS Lett. 2013;587:3875‐3882. 24396870

[27] Zhang Z , Zhu Z , Watabe K , et al. Negative regulation of lncRNA GAS5 by miR‐21. Cell Death Differ. 2013;20:1558‐1568. 23933812 10.1038/cdd.2013.110 PMC3792431

[28] Tian T , Qiu R , Qiu X . SNHG1 promotes cell proliferation by acting as a sponge of miR‐145 in colorectal cancer. Oncotarget. 2018;9:2128‐2139. 29416759 10.18632/oncotarget.23255 PMC5788627

[29] Mahmoudi E , Cairns MJ . miR‐137: an important player in neural development and neoplastic transformation. Mol Psychiatry. 2017;22:44‐55. 27620842 10.1038/mp.2016.150 PMC5414082

[30] Huang YC , Lee CT , Lee JC , et al. Epigenetic silencing of *miR‐137* contributes to early colorectal carcinogenesis by impaired *Aurora‐A* inhibition. Oncotarget. 2016;7:76852‐76866. 27764771 10.18632/oncotarget.12719 PMC5363554

[31] Zhao S , Wang Y , Luo M , Cui W , Zhou X , Miao L . Long noncoding RNA small nucleolar RNA host gene 1 (SNHG1) promotes renal cell carcinoma progression and metastasis by negatively regulating miR‐137. Med Sci Monit. 2018;24:3824‐3831. 29874202 10.12659/MSM.910866 PMC6018379

[32] Peres J , Kwesi‐Maliepaard EM , Rambow F , Larue L , Prince S . The tumour suppressor, miR‐137, inhibits malignant melanoma migration by targetting the TBX3 transcription factor. Cancer Lett. 2017;405:111‐119. 28757416 10.1016/j.canlet.2017.07.018

[33] Ying X , Sun Y , He P . MicroRNA‐137 inhibits BMP7 to enhance the epithelial‐mesenchymal transition of breast cancer cells. Oncotarget. 2017;8:18348‐18358. 28407692 10.18632/oncotarget.15442 PMC5392333

[34] Dong J , Xiao D , Zhao Z , et al. Epigenetic silencing of microRNA‐137 enhances ASCT2 expression and tumor glutamine metabolism. Oncogenesis. 2017;6:e356. 28692032 10.1038/oncsis.2017.59 PMC5541711

[35] Laplante M , Sabatini DM . mTOR signaling in growth control and disease. Cell. 2012;149:274‐293. 22500797 10.1016/j.cell.2012.03.017 PMC3331679

[36] Gulhati P , Cai Q , Li J , et al. Targeted inhibition of mammalian target of rapamycin signaling inhibits tumorigenesis of colorectal cancer. Clin Cancer Res. 2009;15:7207‐7216. 19934294 10.1158/1078-0432.CCR-09-1249 PMC2898570

[37] Guan B , Wu K , Zeng J , et al. Tumor‐suppressive microRNA‐218 inhibits tumor angiogenesis via targeting the mTOR component RICTOR in prostate cancer. Oncotarget. 2017;8:8162‐8172. 28030804 10.18632/oncotarget.14131 PMC5352391

[38] Villanueva A , Chiang DY , Newell P , et al. Pivotal role of mTOR signaling in hepatocellular carcinoma. Gastroenterology. 2008;135:1972‐1983.e11. 1983.e1971‐1911. 18929564 10.1053/j.gastro.2008.08.008 PMC2678688

[39] Schmidt KM , Hellerbrand C , Ruemmele P , et al. Inhibition of mTORC2 component RICTOR impairs tumor growth in pancreatic cancer models. Oncotarget. 2017;8:24491‐24505. 28445935 10.18632/oncotarget.15524 PMC5421865

[40] Sticz T , Molnár A , Márk Á , et al. mTOR activity and its prognostic significance in human colorectal carcinoma depending on C1 and C2 complex‐related protein expression. J Clin Pathol. 2017;70:410‐416. 27729429 10.1136/jclinpath-2016-203913

[41] Laugier F , Finet‐Benyair A , André J , et al. RICTOR involvement in the PI3K/AKT pathway regulation in melanocytes and melanoma. Oncotarget. 2015;6:28120‐28131. 26356562 10.18632/oncotarget.4866 PMC4695048

[42] Gulhati P , Bowen KA , Liu J , et al. mTORC1 and mTORC2 regulate EMT, motility, and metastasis of colorectal cancer via RhoA and Rac1 signaling pathways. Cancer Res. 2011;71:3246‐3256. 21430067 10.1158/0008-5472.CAN-10-4058 PMC3085654

